# Metabolic and Genetic Alterations in Early and Exudative Age-Related Macular Degeneration: Inosine, Amino Acids, and *COL2A1* Gene Variant

**DOI:** 10.3390/ijms27083697

**Published:** 2026-04-21

**Authors:** Akvile Bruzaite, Alvita Vilkeviciute-Petraite, Dzastina Cebatoriene, Dalia Zaliuniene, Ieva Ciapiene, Alina Smalinskiene, Loresa Kriauciuniene, Rasa Liutkeviciene

**Affiliations:** 1 Laboratory of Opthalmology, Neuroscience Institute, Medical Academy, Lithuanian University of Health Sciences, Eiveniu 2, LT-50161 Kaunas, Lithuania; akvile.bruzaite@lsmu.lt (A.B.);; 2 Medical Academy, Lithuanian University of Health Sciences, A. Mickeviciaus St. 9, LT-44307 Kaunas, Lithuania; 3 Department of Ophthalmology, Medical Academy, Lithuanian University of Health Sciences, Eiveniu St. 2, LT-50161 Kaunas, Lithuania; 4 Institute of Cardiology, Lithuanian University of Health Sciences, Sukileliu Av. 15, LT-50103 Kaunas, Lithuania; 5 Institute of Biology Systems and Genetics Research, Lithuanian University of Health Sciences, Eiveniu St. 4, LT-50161 Kaunas, Lithuania

**Keywords:** age-related macular degeneration, gene polymorphism, *COL2A1* gene, collagen type II alpha chain, proline, valine, leucine, inosine

## Abstract

Age-related macular degeneration (AMD) is a complex retinal disease influenced by genetic and metabolic factors. Genetic variants impact disease susceptibility, while alterations in amino acid and purine metabolism are involved in AMD development. This study aimed to examine the association between the *COL2A1* rs1635529 polymorphism and AMD, as well as its relation to specific metabolites. The study comprised 919 participants: 261 with early AMD, 229 with exudative AMD, and 429 controls. DNA was extracted using the salting-out method, and genotyping was performed using real-time PCR. Metabolite levels were analysed with liquid chromatography–mass spectrometry. Statistical analysis was conducted using IBM SPSS Statistics 27.0. Logistic regression revealed that carriers of the GT + TT genotypes had a 1.63-fold higher risk of early AMD (*p* = 0.046). The T allele was also linked to a 1.67-fold elevated risk (*p* = 0.033). No significant associations were observed in exudative AMD. Furthermore, lower leucine levels were noted in exudative AMD patients, and inosine levels were reduced in GT genotype carriers within the early AMD group. The *COL2A1* rs1635529 polymorphism showed a nominal association with early AMD, but not exudative AMD. Differences in leucine and inosine levels were observed, suggesting a potential link between genetic variation and metabolic alterations. These findings indicate possible involvement of collagen-related and metabolic pathways in early disease development; however, the results should be interpreted with caution and require validation in larger studies.

## 1. Introduction

Age-related macular degeneration (AMD) is a progressive, chronic disease of the central retina that causes visual impairment or even irreversible blindness in millions of people worldwide, mainly in developed countries, particularly among Caucasian individuals over the age of 60 and those with blue irises [1,2]. Usually, the disease presents as a gradual loss of central vision: patients report decreased visual acuity, making activities such as reading, driving, and recognising distant objects more difficult [3,4]. Notably, with ageing populations in many countries, more than 20% of the population may have this disorder, and globally, AMD accounts for 8.7% of all blindness [1,5]. The total number of individuals with all types of AMD is approximately 170 million [6]. It has been observed that the incidence rate of AMD varies among racial and ethnic groups, with early manifestations of AMD occurring in 4.2% of Hispanics, 4.6% of Chinese Americans, 5.4% of whites, and 2.4% of blacks [7]. The Lithuanian State Medical Social Examination Commission reported that in 2002, AMD accounted for 13.8% of the primary causes of visual disability [8]. Additionally, the Association of Patients with Retinal Diseases states that there are approximately 30,000 patients with late AMD in Lithuania, with an annual increase of 2000–3000 cases [9].

Clinically, there are two types of AMD: early AMD and late AMD (atrophic/dry and exudative/wet) [10,11]. Early AMD accounts for approximately 80% of cases affecting both eyes, but visual function declines slowly and causes only mild visual disturbances [2]. Late AMD includes atrophic and exudative forms. Atrophic AMD is characterised by progressive retinal pigment epithelium (RPE) atrophy and gradual vision loss over months or years. Exudative AMD is characterised by abnormal vascular proliferation and may lead to sudden and severe central vision loss within days to weeks [12].

The pathological features of AMD range from yellow colloidal deposits (drusen) and RPE changes in early AMD to geographic atrophy and/or choroidal neovascularisation in advanced stages [2]. The main pathological structures involved are the RPE and Bruch’s membrane—a collagen-rich extracellular matrix between the RPE and choroidal vasculature, where drusen accumulate [12]. These polymorphous debris are often the first clinical sign of AMD and an early manifestation of the disease [10].

AMD is a multifactorial disease: its development is affected by both environmental and genetic factors, although its precise pathogenesis remains unclear [7]. One of the primary risk factors is older age [1,7]. Other contributing factors include oxidative stress, lipid peroxidation, inflammation, angiogenesis, and extracellular matrix (ECM) remodelling [13]. Because genetic predisposition plays a key role in AMD development, new genetic markers are continually being investigated [1,14].

Collagen is a key structural protein in the ECM, found in both connective and epithelial tissue membranes [15]. There are two types of collagens based on their structure: fibrillar collagens (types I, II, III, V) and non-fibrillar basement membrane collagen (type IV) [16]. Collagen has various cellular roles, including cell adhesion, which is vital for maintaining tissue structure and function [17]. Most notably, it is a major component of the extracellular matrix [18]. Several studies have linked the *COL2A1* gene to diseases such as chondrogenesis [16], myopia [19], and Stickler syndrome [20], but its connection with AMD has not been explored. Recent research in European and American populations has identified another collagen gene, *COL8A1*, as associated with AMD [21]. The *COL8A1* gene encodes a type VIII collagen alpha chain, with collagen being a central part of basement membranes in the eye [22]. One such structure is Bruch’s membrane, which plays a significant role in the development of AMD [23]. Additionally, *COL10A1*, which encodes the type X collagen alpha chain, has also been linked to AMD [24].

The *COL2A1* gene encodes the alpha-1 chain of type II collagen, a fibrillar collagen that provides structural integrity to connective tissues, is present in the vitreous, and is believed to be part of Bruch’s membrane [25]. The protein comprises 1487 amino acids and has a molecular weight of 141785 Da [26]. A mutation (NCBI SNP rs1635529) has been identified at nucleotide position 48001319 (NC_000012.12: g.48001319), which may influence gene expression. The rs1635529 polymorphism was selected as a candidate variant due to its potential role in regulating *COL2A1* expression and its previously reported associations with ocular disorders [19,20]. Additionally, alterations in collagen-related genes, including *COL8A1* and *COL10A1* [21,24], have been implicated in the pathogenesis of AMD, thereby supporting the relevance of investigating other collagen genes associated with the extracellular matrix, such as *COL2A1*. Furthermore, the relatively common minor allele frequency of rs1635529 in European populations makes this variant appropriate for association analysis within the current study cohort [27]. Given that remodelling of the extracellular matrix is recognised as a significant mechanism in the development of AMD [13], exploring the rs1635529 variant in *COL2A1* may yield further insights into early structural changes in the disease, particularly in Bruch’s membrane.

According to the 1000 Genomes Project, its frequency is 29% in Africans, 37% in Americans, and 15% in Europeans [27].

Although genetic factors play a significant role in the development of AMD, they do not fully explain the disease’s complexity [7,14]. Increasing evidence suggests that metabolic changes are also critically involved in the development and progression of AMD. Metabolomic studies have revealed notable alterations in amino acid, lipid, and nucleotide metabolism in patients with AMD, indicating that metabolic dysregulation may contribute to retinal degeneration [28,29]. These metabolites are closely connected to vital biological processes, including oxidative stress, inflammation, and cellular energy balance, all of which are important in AMD pathophysiology [30]. Therefore, alongside genetic predisposition, studying metabolic factors provides additional insights into the disease’s multifactorial nature and could help identify new biomarkers and therapeutic targets.

Recent studies have underscored the significance of systemic metabolic changes in the pathophysiology of AMD. Metabolomic analyses reveal notable alterations in amino acid, lipid, and nucleotide metabolism in individuals with AMD [28,29]. Among these pathways, purine metabolism has attracted attention due to its roles in energy balance, regulation of oxidative stress, and immune signalling. Inosine, a purine nucleoside produced during the breakdown of adenosine and purine nucleotides, functions as a crucial intermediate in purine metabolism [31]. Inosine also exhibits immunomodulatory and anti-inflammatory properties alongside its metabolic role [32]. Recent microbiome research indicates modifications in purine metabolism in AMD, including changes in inosine-5′-phosphate degradation pathways [33].

Branched-chain amino acids (BCAAs) are among the most abundant essential amino acids and play a vital role in supporting overall human health. Leucine and valine are branched-chain α-amino acids characterised by aliphatic side chains. These amino acids are essential for maintaining nitrogen balance in the body and contribute to skeletal muscle growth and development. Leucine and valine are commonly utilised for their potential roles in supporting muscle recovery, reducing soreness, and modulating immune function [34]. In addition, they partake in many metabolic processes and cellular pathways, highlighting their importance in the complex interactions between diet, health, and ageing [35].

Although leucine strongly activates mechanistic target of rapamycin complex 1 (mTORC1), an amino acid-sensitive protein kinase that plays a key role in regulating metabolism and ageing, leucine restriction has been linked to minimal metabolic benefits, and leucine supplementation has not been proven to significantly affect lifespan. In contrast, studies suggest that valine may be associated with cancer development, inflammation, insulin resistance, and glucotoxicity in mice as well as in human and porcine cell culture models [36]. Valine and its metabolite 3-HIB have also been proposed to directly promote fatty acid and lipid metabolism. As a multifactorial disease, the pathogenesis of AMD is influenced by genetic, environmental, and age-related factors, with abnormalities in lipid metabolism that may, in part, be related to these metabolic processes in AMD [37].

Recent studies suggest that RPE cells prefer proline as a key metabolic substrate and are highly enriched with the proline transporter SLC6A20. Proline supports mitochondrial function, amino acid synthesis, extracellular matrix production, and the maintenance of cellular differentiation [38]. Moreover, proline supplementation has been shown to reduce oxidative damage in cultured RPE cells, while dietary proline provides protection against oxidative stress-induced retinal degeneration in vivo [39]. Interestingly, the neural retina rarely takes up proline directly; instead, it relies on intermediates and amino acids produced from proline metabolism within the RPE. Mutations in genes involved in proline metabolism have been associated with retinal degenerative diseases, and proline supplementation has been reported to reduce vision loss associated with RPE dysfunction [3].

Therefore, this study aims to clarify the interaction between genetic susceptibility and systemic metabolic dysregulation in AMD. We specifically examine the role of the *COL2A1* gene and its rs1635529 polymorphism, alongside changes in key metabolic pathways, including amino acid and purine metabolism. By combining genetic and metabolomic analyses, this work seeks to enhance understanding of AMD pathogenesis and identify new biomarkers that may improve risk assessment and guide future therapeutic strategies.

## 2. Results

The characteristics of the study population are shown in Table 1. The study involved 919 participants (286 men (31.1%) and 633 women (68.9%)), including 490 patients with AMD and 429 controls. No statistically significant differences were found in gender distribution across the three groups (*p* = 0.068). However, significant differences (*p* < 0.001) in age distribution were detected among the study groups using the Kruskal–Wallis H test (Table 1).

Statistical analysis revealed that the distribution of * COL2A1* genotypes was consistent with the Hardy–Weinberg equilibrium in the control group (*p* = 0.157). The frequencies of the *COL2A1* rs1635529 genotypes in patients with early AMD, exudative AMD, and the control group were examined (Table 2 and Table 3). Further analysis demonstrated statistically significant differences (*p* < 0.05) in the genotype distributions of *COL2A1* rs1635529 between the early AMD and control groups (*p* = 0.028) (Table 2).

Statistically significant differences among these groups were also identified by comparing allelic distributions. The rs1635529 polymorphism showed a statistically higher frequency of the T allele in early AMD patients compared to controls (*p* = 0.01) (Table 2).

There were no statistically significant differences between genotypes (GG, GT, and TT) and alleles in patients with exudative AMD and the control group (*p* = 0.099; *p* = 0.278, respectively) (Table 3).

The genotypes of the *COL2A1* gene polymorphism in patients with early AMD, exudative AMD, and the control group were evaluated, with age and gender as covariates. Only the analysis in females revealed a statistically significant difference in the distribution of *COL2A1* rs1635529 genotypes between females with early AMD and controls (*p* = 0.018). Additionally, the rs1635529 polymorphism exhibited a significantly higher frequency of the T allele in females with early AMD compared with controls (*p* = 0.01). (Table 4). We did not find a significant association between rs1635529 and the participants’ age.

Binomial logistic regression analysis was performed to evaluate the ability of rs1635529 to predict the risk of developing early and exudative AMD. In the analysis of early AMD, significant variables were identified: the rs1635529 GT + TT genotypes were linked to a 1.63-fold increased risk of early AMD under the dominant model (OR = 1.630; 95% CI: 1.088–2.645; *p* = 0.046). Also, the results showed that each copy of the T allele at rs1635529 was linked to a 1.67-fold increased risk of early AMD under the additive model (OR = 1.672; 95% CI: 1.042–2.683; *p* = 0.033) (Table 5). The association showed nominal statistical significance before correction; however, after Bonferroni adjustment for the five tested genetic models, the corrected significance threshold was *p* < 0.05/5, and the result no longer remained significant. These findings suggest that the T allele at rs1635529 may be a risk factor for early AMD, although this association should be interpreted with caution due to the lack of significance after multiple testing correction. In contrast, the analysis of exudative AMD did not identify any statistically significant associations.

### 2.1. Metabolite Levels in AMD Patients and the Control Group Subjects

**Figure 1:**
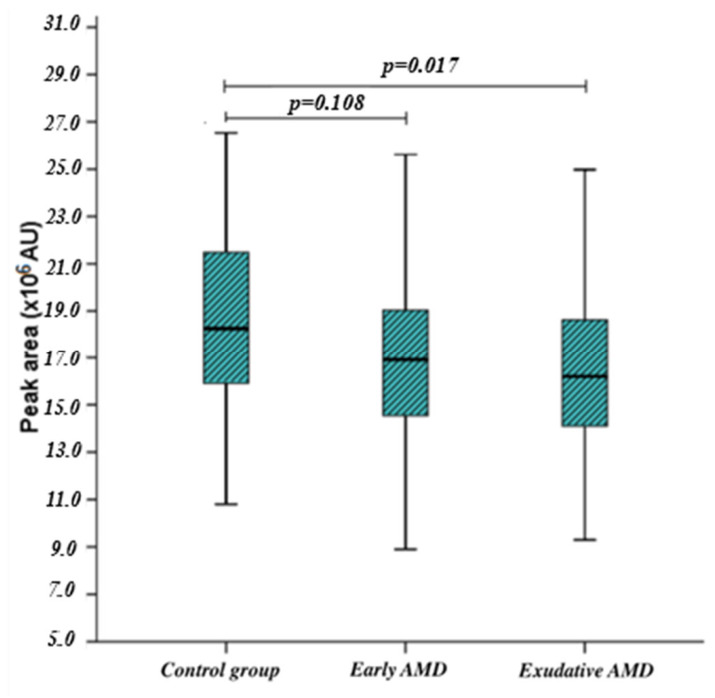
Leucine levels in AMD patients and controls.

Metabolites: proline, valine, leucine, and inosine levels were measured in 88 patients with early AMD, 85 patients with exudative AMD, and 86 control subjects. Serum leucine levels were lower in patients with exudative AMD than in control subjects (*p* = 0.017) (Figure 1). However, after Bonferroni correction for multiple comparisons across the four metabolites (corrected threshold: *p* < 0.05/4), this association did not remain statistically significant.

### 2.2. * COL2A1* rs1635529 Polymorphism and Metabolite Levels

**Figure 2:**
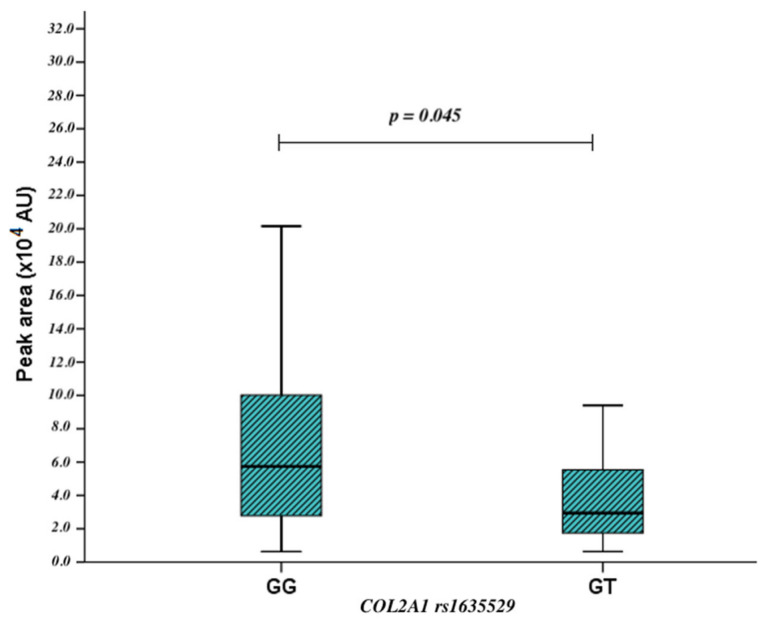
*COL2A1* rs1635529 genotypes and inosine levels in early AMD.

The other objective of our study was to evaluate the associations between the *COL2A1* rs1635529 polymorphism and metabolite levels. We compared proline, valine, leucine, and inosine levels among homozygous wild-type (GG), heterozygous (GT), early exudative AMD, and control groups. Statistically significant differences were revealed only in the early AMD group: inosine levels were lower in GT carriers than in GG (*p* = 0.045) (Figure 2). However, after Bonferroni correction for multiple comparisons across the four metabolites (corrected threshold: *p* < 0.05/4), this association did not remain statistically significant.

## 3. Discussion

Recent research has increasingly focused on novel multifactorial genes associated with age-related macular degeneration (AMD). Among these, COL2A1, which encodes the alpha-1 chain of type II collagen, has become a potential candidate. The complex pathophysiology of AMD may be affected by alterations in the gene responsible for type II collagen, which is believed to be a structural component of Bruch’s membrane.

The structure and permeability of Bruch’s membrane are altered by extracellular matrix remodelling, driven by the degradation and decreased activity of collagen types I, II, and III [13,40,41]. These changes can weaken the membrane’s integrity and promote drusen formation. Consequently, atrophy of the retinal pigment epithelium (RPE) may hinder metabolite transport to photoreceptors, leading to gradual vision loss [7].

Further evidence of COL2A1’s role in AMD development is shown by its expression in Bruch’s membrane. Reduced gene expression may encourage deposit formation and lead to remodelling of the extracellular matrix [42,43]. The biological plausibility of the association between the *COL2A1* rs1635529 polymorphism and early AMD may be explained by the role of type II collagen in maintaining extracellular matrix structure within Bruch’s membrane. Extracellular matrix remodelling is considered an important mechanism in AMD pathogenesis, as degradation and reduced activity of collagen types I, II, and III may alter membrane permeability and promote drusen formation [13,40,41]. Since * COL2A1* is expressed in Bruch’s membrane and contributes to collagen organisation and tissue integrity, genetic variation affecting this gene may contribute to structural alterations of the retinal environment and influence early disease-related changes in AMD [25,42,43]. Since collagen helps sustain tissue integrity and regulates key cellular processes such as adhesion, migration, proliferation, and differentiation, its disruption could be vital in AMD development [13]. In this study, the distribution of *COL2A1* rs1635529 genotypes differed significantly between early AMD patients and controls, with the minor T allele more common in early AMD (*p* = 0.028). Logistic regression analysis indicated that the GT + TT genotypes were associated with an increased risk of early AMD under both the dominant (OR = 1.630; 95% CI: 1.122–2.367; *p* = 0.046) and additive (OR = 1.672; 95% CI: 1.042–2.683; *p* = 0.033) models. Gender differences in AMD susceptibility remain a topic of debate. Colijn et al. reported no significant link between gender and AMD prevalence [44], whereas Rudnicka et al. found a greater risk of exudative AMD in females (OR = 1.2; 95% CI: 1.0–1.5) [45]. In our study, the T allele was notably more frequent among females with early AMD (*p* = 0.01), suggesting a possible gender-specific genetic influence. The variation in genotype distribution between early AMD patients and controls suggests that extracellular matrix remodelling may contribute to early AMD development. However, this represents only one component of a complex, multifactorial disease. The observed odds ratios indicate a modest effect size, suggesting that this polymorphism represents only one of multiple genetic factors contributing to AMD susceptibility.

This is the first study, to our knowledge, to evaluate the * COL2A1* rs1635529 polymorphism in AMD within a Lithuanian population. Although this polymorphism has been investigated in various ocular disorders, no comparable research on AMD was identified in existing databases. While Metlapally et al. demonstrated significant associations (*p* < 0.045) and examined additional polymorphisms in a European cohort, Wang et al. found no link between rs1635529 and severe myopia in a Chinese cohort [46]. These differences may stem from demographic or ethnic variations. *COL2A1* polymorphisms have also been linked to systemic disorders beyond ocular diseases. Mutations in *COL2A1* are known to cause skeletal abnormalities such as chondrogenesis type II, hypochondrogenesis, and spondyloepiphyseal dysplasia congenita [26], while Hoornaert et al. demonstrated its involvement in type I diabetes mellitus with progressive arthropathy [20]. Although the role of specific polymorphisms in the development and progression of AMD remains uncertain, despite the identification of approximately 20 chromosomal regions associated with the disease [7], our findings suggest that the *COL2A1* rs1635529 polymorphism may represent a modest genetic susceptibility factor for early AMD. Nevertheless, further research is required to evaluate its potential as a predictive biomarker.

Aside from genetic factors, this study investigated inosine as a potential metabolite because of its role in purine metabolism and immune regulation. Inosine, derived from adenosine and other purine nucleotides, can be further degraded to hypoxanthine and uric acid, linking nucleotide metabolism to oxidative stress and redox homeostasis [32]. Changes in inosine levels might indicate systemic metabolic and inflammatory imbalances. Microbiome research suggests that purine metabolic pathways, including inosine-5′-phosphate degradation, could be altered in patients with AMD, implying interactions between microbial metabolism and the host immune response [33]. These findings support a role for purine metabolism in AMD-related inflammation. Although initially identified in geographic atrophy, our results extend these observations to early and exudative AMD, indicating that disruptions in purine metabolism may occur at various stages of the disease. Given the key roles of inflammation, complement activation, and oxidative stress in AMD, purine metabolites such as inosine might reflect fundamental immune-metabolic processes [30]. Proline metabolism also plays an important role in retinal physiology. Experimental studies have demonstrated that proline is utilised more rapidly than glucose by RPE cells and supports retinal energy metabolism. Proline-derived metabolites are transported to the retina, as confirmed in vivo [38].

Furthermore, dietary proline has been shown to protect RPE cells from oxidative stress and improve visual function, while age-related changes in the choroid–RPE–retinal complex contribute to the development of AMD. Elevated proline levels observed in our study may therefore reflect systemic metabolic changes. This is supported by genome-wide association studies linking the proline transporter SLC6A20 to AMD, as well as evidence that disruptions in proline metabolism can cause retinal degeneration [47]. Other metabolomic studies have reported reduced levels of amino acids (valine, leucine, isoleucine, phenylalanine, tyrosine) and citrate in patients with AMD, suggesting altered metabolic or microbiome-related processes [48]. Mendez et al. identified associations between branched-chain amino acid metabolites and impaired dark adaptation, while Thee et al. reported decreased amino acid levels and increased ketone bodies in exudative AMD [49,50].

Although major systemic diseases known to significantly affect metabolic profiles (such as diabetes mellitus, malignant tumours, chronic infectious diseases, connective tissue disorders, and post-transplantation status) were excluded according to the study protocol, some potential confounding factors may still have influenced metabolite levels. Dietary habits, use of nutritional supplements (including AREDS formulations), smoking status, medication use, and previous anti-VEGF treatment were not fully controlled for in this study. Therefore, these factors should be considered when interpreting the metabolomic findings.

Overall, these findings support the idea that AMD is a complex disease caused by multiple factors, including interactions between genetic and metabolic elements. The *COL2A1* rs1635529 polymorphism may contribute to early AMD susceptibility, possibly through mechanisms related to extracellular matrix remodelling [13,29,30,31,32]. Simultaneously, metabolic disturbances—such as alterations in amino acids, proline, and purine metabolism-appear to play a significant role [40,42,46]. Proline supports RPE energy metabolism, whereas inosine signals inflammatory and oxidative processes [33]. Microbiome-related changes in purine metabolism might also contribute to disease development [34]. In summary, the interaction of genetic predisposition, metabolic imbalance, oxidative stress, and inflammation is central to AMD formation [31].

## 4. Materials and Methods

### 4.1. Ethics

The research was carried out in line with the principles set out in the Declaration of Helsinki. Ethical approval was obtained from the Kaunas Regional Biomedical Research Ethics Committee at the Lithuanian University of Health Sciences (approval No. BE-2-/48). All analyses were conducted at the Laboratory of Ophthalmology, the Neuroscience Institute, Lithuanian University of Health Sciences (LUHS).

### 4.2. Establishment of the Study Groups

A total of 919 subjects were genotyped, including patients with early AMD (n = 261), patients with exudative AMD (n = 229), and healthy controls (n = 429). All study participants were of Lithuanian origin, as determined by self-reported ethnicity, and were recruited from the same geographical region. Thus, the study population is relatively ethnically homogeneous among Northern Europeans, reducing the likelihood of population stratification bias. The relatively homogeneous ethnic composition of the study population also reduces the potential influence of population stratification on the genetic analysis.

### 4.3. Inclusion Criteria for the AMD Group

- Patients diagnosed with early or late AMD who signed informed consent to participate in the study were included.
- Males and females aged between 50 and 99 years.
- The diagnosis of exudative AMD was based on the results of an optical coherence tomography examination.
- Fluorescein angiograms were performed if necessary. The classification system for AMD, proposed by the Age-Related Eye Disease Study Research Group in 2001, was utilised.

### 4.4. Exclusion Criteria for Patients with AMD Group

- Unrelated eye disorders, such as high refractive error, cloudy cornea, lens opacity (nuclear, cortical, or posterior subcapsular cataract), except minor opacities, keratitis, acute or chronic uveitis, glaucoma, or diseases of the optic nerve.
- Systemic illnesses, such as diabetes mellitus, malignant tumours, systemic connective tissue disorders, chronic infectious diseases, or conditions following organ or tissue transplantation.
- Ungraded colour fundus photographs resulting from obscuration of the ocular optic system or due to poor photograph quality.

### 4.5. Inclusion Criteria for Control Group Subjects

- Ophthalmologically healthy subjects.
- Males and females aged from 19 to 99 for SNP analysis and from 50 to 99 for metabolites analysis.
- No chronic infectious or non-infectious diseases.
- Signed informed consent to participate in the research.

### 4.6. Exclusion Criteria for Control Group Subjects

- Any eye disorders or ungraded optical coherence tomography caused by obstruction of the ocular optics system.
- Systemic illnesses, such as malignant tumours, systemic connective tissue disorders, chronic infectious diseases, or conditions following organ or tissue transplantation.
- Use of epileptic and sedative drugs.

### 4.7. Ophthalmological Evaluation

All study subjects were evaluated using a slit-lamp biomicroscope to assess corneal and lenticular transparency. Classification and grading of lens opacities were performed according to the Lens Opacities Classification System III. During each examination, intraocular pressure was measured. Pupils were dilated with 1% tropicamide, after which fundoscopy was carried out with a direct monocular ophthalmoscope and slit-lamp biomicroscope using a double aspheric lens of +78 dioptres. The results of eye examinations were recorded on standardised forms. For detailed analysis of the macula, stereoscopic colour fundus photographs centred at 45° and 30° to the fovea were obtained with a Visucam NM Digital camera (Carl Zeiss Meditec AG, Jena, Germany). All AMD patients underwent optical coherence tomography (OCT), and fluorescein angiography was performed in patients suspected of having late AMD after OCT.

The AMD classification system based on the Age-Related Eye Disease Study (AREDS classification) was utilised: early mild AMD included a combination of several small drusen and some intermediate drusen (63–124 μm in diameter), or retinal pigment epithelial (RPE) abnormalities. Early intermediate AMD was characterised by the presence of extensive intermediate drusen and at least one large (≥125 μm diameter) druse, or geographic atrophy not affecting the centre of the fovea. Advanced AMD was defined by geographic atrophy involving the fovea and/or any features of neovascular AMD (AREDS classification).

### 4.8. DNA Extraction and Genotyping

Blood was collected from each participant and stored in ethylenediaminetetraacetic acid (EDTA) tubes. Genomic DNA was extracted from peripheral blood leukocytes using the salt-out method. UV spectrophotometry (Agilent Technologies, Santa Clara, CA, USA, Cary 60 UV-Vis) was employed to determine DNA concentration and purity index in each blood sample, based on the absorbance ratio at 260/280 nm. All samples showed a purity index of 1.8 to 2.0. The rs1635529 SNP of the *COL2A1* gene was examined using TaqMan^®^ Genotyping assays (Applied Biosystems, Foster City, CA, USA). The Applied Biosystems 7900HT Real-Time Polymerase Chain Reaction System was utilised for SNP detection. Appropriate Real-Time PCR mixtures were prepared following the manufacturer’s protocol for SNP determination. Allelic discrimination and standard curves were generated using “SDS 2.3” software (Applied Biosystems, Foster City, CA, USA). The software identified individual genotypes of the polymorphism based on the fluorescence intensities of the reporter dyes VIC and FAM.

### 4.9. Metabolite Profiling

Blood samples were mixed with an ice-cold methanol–water solution (final methanol concentration 50%) to extract metabolites, precipitate proteins, and inhibit enzymatic activity. Internal standards (HEPES and PIPES) were added to the mixture. The samples were then filtered through a 10 kDa cutoff filter to remove high-molecular-weight components. Prepared samples were subsequently shipped for metabolomic analysis.

The extracts were evaporated to concentrate metabolites and replace the solvent. Metabolite separation was performed using a ZIC-HILIC high-performance liquid chromatography (HPLC) column optimised for polar compounds, and detection was carried out using an Orbitrap mass spectrometer in both positive and negative ionisation modes.

HPLC–mass spectrometry data were processed using MZmine 2 software (version 2.21). Following peak detection in MZmine 2, isotopic peaks were systematically eliminated, and peak lists were subsequently aligned across samples utilizing precise mass (*m*/*z*) measurements and retention time to ensure measurement comparability. Metabolite identification was corroborated through the matching of retention times and *m*/*z* values against reference standards. Peak areas were normalized through the signal intensities of spiked internal standards (HEPES and PIPES) to enhance analytical consistency across samples.

### 4.10. Statistical Analysis

Statistical analyses were conducted using IBM SPSS/W 27.0 software (Statistical Package for the Social Sciences for Windows, Inc., Chicago, IL, USA). Categorical variables are presented as counts (percentages), while continuous variables are expressed as median (interquartile range, IQR), as appropriate. Hardy–Weinberg equilibrium was assessed using the chi-square test. Genotype distributions between groups were compared using the chi-square test or Fisher’s exact test. Normality was evaluated using the Shapiro–Wilk and Kolmogorov–Smirnov tests. Age differences between groups were assessed using the Kruskal–Wallis test. A *p*-value < 0.05 was considered statistically significant.

Metabolite concentrations are presented as raw peak area values (×10^6^ arbitrary units). Due to the non-normal distribution of the data, group comparisons were performed using non-parametric tests (Mann–Whitney U test or Kruskal–Wallis test, as appropriate). To account for multiple comparisons across the four analyzed metabolites, Bonferroni correction was applied, and the threshold for statistical significance was set at *p* < 0.0125 (0.05/4).

Binary logistic regression analysis was performed to assess the association between genotype and the risk of early and exudative AMD, with results expressed as odds ratios (ORs) and 95% confidence intervals (CIs), adjusted for age and sex. To control for multiple testing across the five genetic models, Bonferroni correction was applied, and the threshold for statistical significance was set at *p* < 0.01 (0.05/5).

## 5. Conclusions

The * COL2A1* rs1635529 polymorphism showed a nominal association with early AMD and may represent a modest genetic susceptibility factor, with the T allele potentially contributing to increased susceptibility, particularly among females. No significant association was observed with exudative AMD. In addition, AMD patients exhibited altered metabolite levels, particularly leucine and inosine, suggesting a potential link between genetic variation and metabolic alterations. These findings may indicate the involvement of collagen-related and metabolic pathways in the early stages of AMD development. However, given the modest strength of the associations and potential limitations related to sample size and multiple testing, the results should be interpreted with caution. Further studies in larger, independent cohorts are required to confirm these observations and to clarify the underlying biological mechanisms.

## Figures and Tables

**Table 1:** Demographic characteristics of the study population.

Characteristics	Group			*p* Value
	Early AMD n = 261	Exudative AMD n = 229	Control n = 429	
Males, n (%) Females, n (%)	86 (33.0) 175 (67.0)	82 (33.8) 147 (64.2)	118 (27.5) 311 (72.5)	0.068
Age, mean (SD)	70.57 (10.13)	75.02 (8.51)	49.34 (19.68)	**<0.001**

AMD—age-related macular degeneration; SD—standard deviation. *p*-value—significance level. Note: Significant results are indicated in bold.

**Table 2:** Genotype and allele distributions of *COL2A1* rs1635529 in early AMD and control groups.

SNP	Genotype/Alleles	Controls, n (%) (n = 429)	Early AMD, n (%) (n = 261)	HWE *p*-Value	*p*-Value
rs1635529	GG GT TT	354 (82.5) 74 (17.2) 1 (0.2)	194 (74.3) 65 (24.9) 2 (0.8)	0.157	**0.028**
	G T	782 (91.1) 76 (8.9)	453 (86.8) 69 (13.2)		**0.01**

SNP—single-nucleotide polymorphism; HWE—Hardy–Weinberg equilibrium; AMD—age-related macular degeneration. *p*-value—significance level. Note: Significant results are indicated in bold.

**Table 3:** Genotype and allele distributions of *COL2A1* rs1635529 in exudative AMD and control groups.

SNP	Genotype/Alleles	Controls, n (%) (n = 429)	Exudative AMD, n (%) (n = 229)	HWE *p*-Value	*p*-Value
rs1635529	GG GT TT	354 (82.5) 74 (17.2) 1 (0.2)	184 (80.3) 41 (17.9) 4 (1.7)	0.157	0.099
	G T	782 (91.1) 76 (8.9)	409 (89.3) 49 (10.7)		0.278

SNP—single-nucleotide polymorphism; HWE—Hardy–Weinberg equilibrium; AMD—age-related macular degeneration. *p*-value—significance level.

**Table 4:** Genotype and allele distributions of *COL2A1* rs1635529 in early AMD and control females.

SNP	Genotype/Alleles	Control Females, n (%) (n = 311)	Early AMD Females, n (%) (n = 175)	*p*-Value
rs1635529	GG GT TT	257 (82.6) 54 (17.4) 0 (0.0)	127 (72.6) 47 (26.9) 1 (0.6)	**0.018**
	G T	568 (91.3) 54 (8.7)	301 (86.0) 49 (14.0)	**0.01**

SNP—single-nucleotide polymorphism; AMD—age-related macular degeneration. *p*-value—significance level. Note: Significant results are indicated in bold.

**Table 5:** Analysis of *COL2A1* rs1635529 using binary logistic regression in patients with early AMD and the control groups.

Model	Genotype	OR * (95% CI)	*p* Value
Codominant	GT vs. GG TT vs. GG	1.587 (0.978–2.577) 20.351 (0.296–1399.063)	0.062 0.163
Dominant	GT + TT vs. GG	1.633 (1.088–2.645)	**0.046**
Recessive	TT vs. GG + GT	18.045 (0.269–1209.283)	0.178
Overdominant	GT vs. GG + TT	1.569 (0.967–2.545)	0.068
Additive	T	1.672 (1.042–2.683)	**0.033**

OR—odds ratios; *—OR adjusted by age; CI—confidence intervals. *p*-value—significance level. Note: Significant results are indicated in bold.

## Data Availability

The datasets used and/or analysed during the current study are available from the corresponding author upon reasonable request.
